# Pigment epithelium‐derived factor peptide promotes limbal stem cell proliferation through hedgehog pathway

**DOI:** 10.1111/jcmm.14364

**Published:** 2019-05-08

**Authors:** Nai‐Wen Fan, Tsung‐Chuan Ho, Cheng‐Wen Wu, Yeou‐Ping Tsao

**Affiliations:** ^1^ Department of Ophthalmology Taipei Veterans General Hospital Taipei Taiwan; ^2^ Institute of Clinical Medicine National Yang‐Ming University Taipei Taiwan; ^3^ Department of Medical Research Mackay Memorial Hospital Taipei Taiwan; ^4^ Institute of Biomedical Sciences Academia Sinica Taipei Taiwan; ^5^ Institute of Microbiology and Immunology National Yang Ming University Taipei Taiwan; ^6^ Institute of Biochemistry and Molecular Biology National Yang Ming University Taipei Taiwan; ^7^ Department of Ophthalmology Mackay Memorial Hospital Taipei Taiwan

**Keywords:** 44‐mer, limbal stem cell deficiency, limbal stem cells, PEDF, Sonic Hedgehog, STAT3

## Abstract

Expansion of limbal epithelial stem cells (LSCs) is crucial for the success of limbal transplantation. Previous studies showed that pigment epithelium‐derived peptide (PEDF) short peptide 44‐mer could effectively expand LSCs and maintain them in a stem‐cell state, but the mechanism remained unclear. In the current study, we found that pharmacological inhibition of Sonic Hedgehog (SHh) activity reduced the LSC holoclone number and suppressed LSC proliferation in response to 44‐mer. In mice subjected to focal limbal injury, 44‐mer facilitated the restoration of the LSC population in damaged limbus, and such effect was impeded by the SHh or ATGL (a PEDF receptor) inhibitor. Furthermore, we showed that 44‐mer increased nuclear translocation of Gli1 and Gli3 in LSCs. Knockdown of *Gli1* or *Gli3* suppressed the ability of 44‐mer to induce cyclin D1 expression and LSC proliferation. In addition, ATGL inhibitor suppressed the 44‐mer‐induced phosphorylation of STAT3 at Tyr705 in LSC. Both inhibitors for ATGL and STAT3 attenuated 44‐mer‐induced SHh activation and LSC proliferation. In conclusion, our data demonstrate that SHh‐Gli pathway driven by ATGL/STAT3 signalling accounts for the 44‐mer‐mediated LSC proliferation.

## 1 INTRODUCTION

Corneal integrity and transparency are indispensable for normal vision. The corneal epithelium is the outmost layer of corneas and is constantly renewed from limbal epithelial stem cells (LSCs).^1^ Human LSCs have been identified at the basal epithelial layer of the limbus, anatomically located at the boundary of the cornea and the conjunctiva.^2^ Destruction of the limbus potentially leads to visual impairment because of conjunctivalization of the corneas, corneal neovascularization (NV), scaring, chronic inflammation or recurrent/persistent corneal epithelial defects.^3^

Transplantation of limbal tissue/stem cells is a prevalent therapeutic approach for patients with LSC deficiency (LSCD).^4^, ^5^, ^6^, ^7^ Although combination with amnion membrane grafting has substantially improved the outcome, the long‐term success rate for advanced LSCD remains low because of the lack of proper regeneration of LSCs.^8^, ^9^, ^10^, ^11^ Therefore, promoting expansion of stem cell population can increase the success of ocular reconstruction.^6^, ^11^

Pigment epithelium‐derived factor (PEDF) is a 50‐kDa secreted glycoprotein with multiple biologic effects on various types of cells.^12^ The amino acid positions Val78‐Thr121 of human PEDF (termed 44‐mer) is responsible for neurotrophic and mitogenic activity.^13^, ^14^, ^15^ 44‐mer has been proven to be able to promote LSC proliferation and meanwhile maintain a stem‐cell state.^14^ Further in vivo studies have confirmed that the 44‐mer effectively repopulates LSCs in damaged limbus in rabbits.^16^, ^17^ These encouraging results suggest the potential role of 44‐mer in treating LSCD or increasing the survival of limbal grafts. However, the mechanism of 44‐mer‐mediated LSC self‐renewal has yet to be elucidated.

Sonic Hedgehog (SHh) signalling pathway is critical for maintaining and supporting stem cell properties in various tissues.^18^, ^19^, ^20^, ^21^ Secreted SHh ligands act on responding cells by turning on an intracellular signalling pathway that induces Gli transcription factors^18^. The active stage of the SHh pathway is tightly controlled and regulated by the integrating pathways to keep cellular processes balanced. Corneal wounding induced transient up‐regulation of SHh ligands and expression of Gli3 in the limbus, suggesting that LSC behaviour is regulated by the SHh pathway.^22^ In this study, we investigated whether SHh signalling pathway could be induced by 44‐mer and promote the expansion of LSCs. We found that 44‐mer‐induced Gli1 and Gli3 expressions to promote LSC proliferation, and such effect was suppressed by ATGL and STAT3 inhibitor.

## 2 MATERIALS AND METHODS

### 2.1 Chemicals and antibodies

Antibodies used in this study were △Np63α (Biolegend, San Diego, CA.), Lrig‐1 (ab36707, Abcam, Cambridge, MA), CK19 (ab15463; Abcam, Cambridge, MA) and CK12 (BS4625R; Bioss, Woburn, MA). Antibodies against Patched (Ptch), Smoothened (SMO), Gli1, Gli3, Gli3‐R, histone H1 and cyclin D1 were purchased from GeneTex. Antibody against phospho‐STAT3 (Tyr705) was purchased from Cell Signaling Technology (Beverly, MA). All the fluorescent dye‐conjugated secondary antibodies were from BioLegend (San Diego, CA). Atglistatin (530151), STAT3 Inhibitor Peptide (s73096) and STAT3 Inhibitor V (573099) were from Calbiochem (La Jolla, CA). HPI4, cyclopamine, DAPT and 5‐bromo‐2’‐deoxyuridine (BrdU), Hoechst 33258 dye and formalin were from Sigma‐Aldrich (St. Louis, MO). Dispase II and epidermal growth factor were from Roche (Indianapolis, IN). Recombinant human SHh was from Peprotech (NJ). Dexamethasone (5 mg/1 mL) was from Taiwan Biotech Co. (Taoyuan, Taiwan). Haematoxylin and eosin (H&E) dyes were purchased from Merck (Rahway, NJ). The 44‐mer (Val78‐Thr121) and 18‐mer (Glu97‐Ser114; a control peptide) were synthesized, modified by acetylation of the NH_2_ termini and amidation of the COOH termini for stability, and characterized by mass spectrometry (>90% purity) at GenScript (Piscataway, NJ). PEDF were reconstituted in DMSO as a stock solution (5 mmol/L).

### 2.2 Animals

New Zealand albino rabbits (3.0‐3.5 kg, 6 months old) and 3‐5‐month‐old Balb/c mice were used. All procedures were approved by the Mackay Memorial Hospital Review Board for animal investigation and were conducted in accordance with the ARVO statement for the Use of Animals in Ophthalmic and Vision Research.

### 2.3 Limbal stem cell culture

LSCs were isolated from rabbits and used for cell‐suspension culture, colony‐forming efficiency (CFE) and BrdU labelling assay as described previously.^14^ Briefly, the limbal rings were washed in phosphate‐buffered saline containing 50 μg/mL gentamicin. After the iris and excessive sclera were removed, the limbal rings were exposed to dispase II (1.2 IU/mL in Hanks’ balanced salt solution free of Mg^2+^ and Ca^2+^) at 4°C for 16 hours. The loosened epithelial sheet was harvested with a cell scraper and separated into single cells by treating with 0.5 mL trypsin (0.25% and 0.01% EDTA) for 15 minutes at 37°C with gental shaking. Cells were transferred to 9 mL of 10% FBS/DMEM/F‐12 medium and were then collected by centrifugation (400 g for 5 minutes). LSCs were cocultured with MMC‐treated NIH‐3T3 fibroblast feeder cells located within the transwell (0.4 μm pore, BD Biosciences, Bedford, MA). For passage, near confluent cells were harvested with 0.25% trypsin and then 1  10^5^ subcultured cells were cultured in the respective medium described above.

### 2.4 Colony‐forming efficiency

Approximately 1 x 10^3^ LSCs were seeded in a 3.8‐mm^2^ dish and cocultured with MMC‐treated NIH‐3T3 feeder cells located within the transwell. The medium was changed every 2‐3 days. At 10 days, colonies were fixed by 4% paraformaldehyde (room temperature for 1 hour) for immunostaining and crystal violet staining. The CFE (%) was calculated using the following formula: number of colonies formed/number of cells plated × 100%.

### 2.5 Western blotting

Cell lysis and SDS PAGE were performed as described previously.^23^ NE‐PER nuclear and cytoplasmic extraction kit (Pierce, Rockford, IL) was used to separate total cell lysate into cytoplasmic and nuclear fractions according to the manufacturer's instructions. Each cellular fraction was then resolved by SDS‐polyacrylamide gel electrophoresis, electrotransferred to polyvinylidene difluoride membranes (Millipore, Bedford, MA), and processed for immunoblot analysis. Antibodies used in the immunoblot study were cyclin D1, Ptch, SMO, Gli1, Gli3, Gli3‐R, histone H1 and phospho‐STAT3 (1:1000 dilution). Proteins of interest were detected using the appropriate IgG‐HRP secondary antibody and ECL reagent. X‐ray films were scanned on a Model GS‐700 imaging densitometer (Bio‐Rad Laboratories, Hercules, CA) and analysed with Labworks 4.0 software. Blots from at least three independent experiments were used for quantification.

### 2.6 Quantitative real‐time PCR

Experiments were performed as described previously.^23^ The sequences of the PCR primers were rabbit *Gli1* (accession number: XM_017350480.1) sense, 5′‐ CTTCAAGGCCCAGTACATGC‐3′, anti‐sense, 5′‐TCGAGGCGTGAGTATGACTT‐3′; rabbit *Gli3* (XM_017344449.1) sense, 5′‐ ACAGGCGAGAAGCCTCATAA‐3′, anti‐sense, 5′‐ CAACCTTCGTGCTCACAGA C‐3′; and rabbit *GAPDH* (NM_001082253;) sense, 5′‐TCTGGCAAAGTGGATGTTGT‐3′, anti‐sense, 5′‐GTGGGTGGAATCATACTGGA‐3′. The cycle threshold (Ct) values of the PCR product and a *GAPDH* (Glyceraldehyde 3‐phosphate dehydrogenase) control mRNA were used to calculate relative quantities of mRNA.

### 2.7 siRNA transfection

LSCs were transfected with 10 nmol/L of siRNA targeting *Gli1* or *Gli3* genes (listed in Table 1; Stealth RNAi™ siRNA duplexes, Invitrogen) for 48 hours using the Lipofectamine RNAi MAX transfection kit according to the manufacturer's protocol (Invitrogen, USA). Scramble siRNA (sc‐37007, Santa Cruz Biotechnology) that did not specifically target any gene were used as control.

**Table 1:** Sequence of siRNA to Gli1 and Gli3 in rabbits

siRNA	Sequence (5′‐3′)	Accession number
Gli1‐1	F: CCA GUG UCC UCG ACU UGA ACA UUA U R: AUA AUG UUC AAG UCG AGG ACA CUG G	XM_017350480.1
Gli1‐2	F: UAG AGU UGA GGA AUU GCG UCU CUC C R: GGA GAG ACG CAA UUC CUC AAC UCU A	
Gli3‐1	F: CAC GUG CCU UCU GCC UUA UCU AGU A R: UAC UAG AUA AGG CAG AAG GCA CGU G	XM_017344450.1
Gli3‐2	F: GGA CCA AAU GGA UGG AGC ACG UAA A R: UUU ACG UGC UCC AUC CAU UUG GUC C	

To evaluate the influence of *Gli1* and *Gli3* knockdown on LSC proliferation, LSCs (2 × 10^3^/well) were seeded in 96‐well cell culture plate (Costar; cat. no. 3599) one day before transfection. Forty‐eight hours after siRNA transfection, cells were maintained in serum‐free DMEM/F12 for 2 hours and then stimulated by 44‐mer (10 µmol/L) for 24 hours. The level of LSC proliferation was evaluated by Cell Proliferation Assay Kit based on the amounts of nuclear dye binding (BioVision; Catalog # K307‐1000), according to the company's instruction. All assays were performed in triplicate, and the experiment was independently performed for three times.

### 2.8 Partial limbal injury

Mice were anaesthetized by intraperitoneal injection of a mixture of zoletil (6 mg/kg) and xylazine (3 mg/kg). One drop of 0.5% proparacaine hydrochloride (Alcaine; Alcon,Fort Worth, TX) was given before ocular procedures. The epithelium of the inferior 120 degree limbus was removed with a 0.5 mm metal burr (Rumex international Co, Clearwater, FL),^24^ 0.75 mm into the cornea and 0.75 mm into the conjunctiva in the experimental eye.

The 44‐mer was reconstituted in DMSO to a final concentration 100 μmol/L. At the end of limbal surgery, a separated dose of 10 μL of 44‐mer (100 μmol/L) mixed with 90 μL of dexamethasone was injected into the upper and lower conjunctival fornix. 10 μL of DMSO mixed with 90 μL of dexamethasone served as a control. Mice were killed at 2 weeks.

To study the role of signalling pathways, subconjunctival injection of 10 μL 44‐mer and 85 μL dexamethasone mixed with various inhibitors was performed: HPI4 (SHh inhibitor, 5 μL of 500 μmol/L) and Atglistatin (ATGL inhibitor, 5 μL of 500 μmol/L). 10 μL 44‐mer and 85 μL dexamethasone with 5 μL DMSO served as a control. Mice were killed at 2 weeks and the eyeballs were harvested for immunofluorescence (IF) staining.

### 2.9 Immunofluorescence

Deparaffinized tissue sections (5 μm) or 4% paraformaldehyde‐fixed LSCs were blocked with 10% goat serum and 5% BSA in PBS containing 0.1% Tween 20 for 1 hour. Staining was performed with primary antibodies against CK12 (1:200 dilution), CK19, △Np63α, Lrig1, Gli1, Gli3, BrdU (all 1:100 dilution) for 2 hours at 37°C, followed by incubation with appropriate rhodamine‐ or FITC‐conjugated donkey IgG (1:500 dilution) for 1 hour at room temperature. Images were acquired with a Zeiss epifluorescence microscope and a charge‐coupled device camera. Photographs were taken with the Zeiss Axiovision version 3.1 software (Carl Zeiss MicroImaging GmbH, Jena, Germany).

### 2.10 Organ culture

To evaluate the expressions of Gli1 and Gli3 in the nucleus of remaining LSCs after partial limbal injury, murine eyes were enucleated and placed in a 24‐well culture plate containing 2 mL LSC culture medium^14^ supplemented with 10 µmol/L 44‐mer for 2 hours at 37°C. LSC culture medium supplemented with DMSO served as the control. Each globe was fixed in 4% paraformaldehyde in 0.1 mol/L phosphate buffer (PH 7.4) for 48 hours at 4°C and then embedded in paraffin.

### 2.11 Statistical analysis

Results were presented as mean ± SD. The statistic significances of the experimental results were assessed by Student's *t* test using SPSS version 18.0 (SPSS Inc, Chicago, IL). A two‐tailed *P *< 0.05 was considered statistically significant. Statistical significance was as follows: *P* < 0.05 (*), *P* < 0.01 (**), and *P* < 0.001 (***).

## 3 RESULTS

### 3.1 SHh signalling promotes LSC proliferation

**Figure 1:**
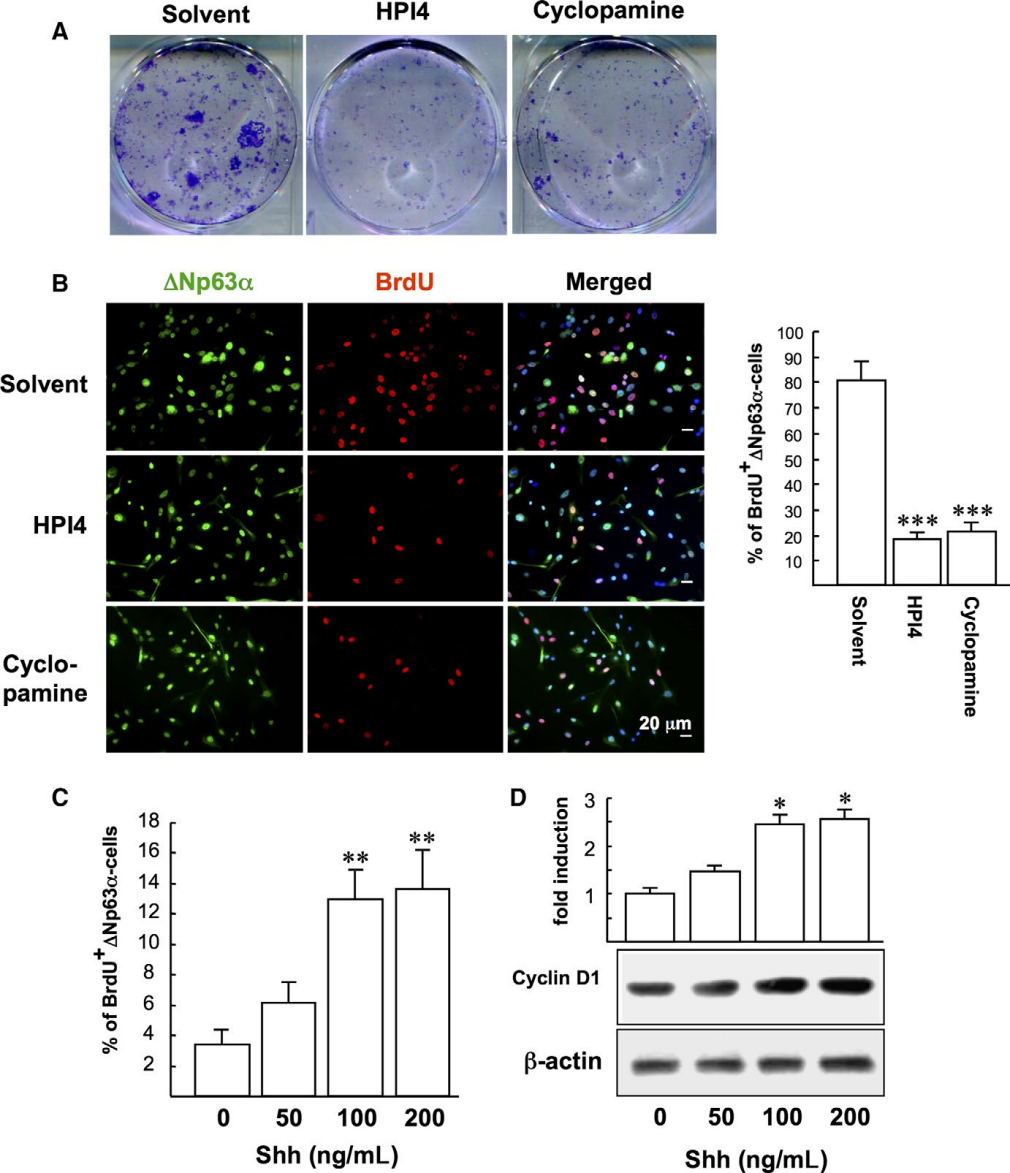
Inhibition of Sonic Hedgehog (SHh) signalling reduces the proliferation of limbal stem cells (LSC). (A) Colony‐forming efficiency assay. LSC were cultured in the presence of 1 µmol/L HPI4, cyclopamine or solvent control (DMSO). After 10 days, colonies were observed by staining the plates with 0.5% crystal violet. Three independent experiments were performed. (B) LSC were treated with 10 µmol/L HPI4 or cyclopamine and proliferation was measured by BrdU labelling for 24 h. LSC (∆Np63α; *green*) and BrdU (*red*) were detected by immunofluorescence microscopy (original magnification, ×400). A representative picture of three independent experiments is shown. Ten randomly selected fields in each group were photographed, and the percentage of BrdU and ∆Np63α‐double positive cells (pale pink) per total ∆Np63α‐positive cells was calculated. ***P < 0.0002 vs solvent‐treated cells. (C) LSC were treated with SHh (0‐200 ng) for 24 h. At the end of the incubation period, cell proliferation was measured by 2‐hour BrdU labelling. **P < 0.004 vs untreated cells. (D) Western blot analysis of the expression of cyclin D1 in LSC treated as described above. Blotting of β‐actin serves as a loading control. Representative blots and densitometric analysis with the SD from three independent experiments are shown. *P < 0.05 vs untreated cells

We first examined the influence of SHh signalling on LSC clonogenicity by exposing LSCs to SHh inhibitors (HPI4 or cyclopamine) for 10 days. The clonogenicity of LSC was significantly suppressed by SHh inhibitors compared to control (Figure 1A). In addition the BrdU proliferation assay showed that HPI4 and cyclopamine significantly reduced the proliferation of ∆Np63α‐positive LSCs compared to control (17.8 ± 2.7% and 21.3 ± 3.2% vs 80.5 ± 7.1%), suggesting that suppression of SHh signalling impaired LSC proliferation (Figure 1B). To confirm our findings, LSCs were further treated with recombinant human SHh at various concentrations (50, 100 and 200 ng/mL) for 24 hours before 2 hours of BrdU labelling. SHh was found to increase LSC proliferation (1.8‐4.0‐fold; Figure 1C) and cyclin D1 expression (1.4‐2.6‐fold; Figure 1D) in a dose‐dependent manner.

### 3.2 44‐mer‐induced LSC proliferation depends on Sonic Hedgehog (SHh) pathway

**Figure 2:**
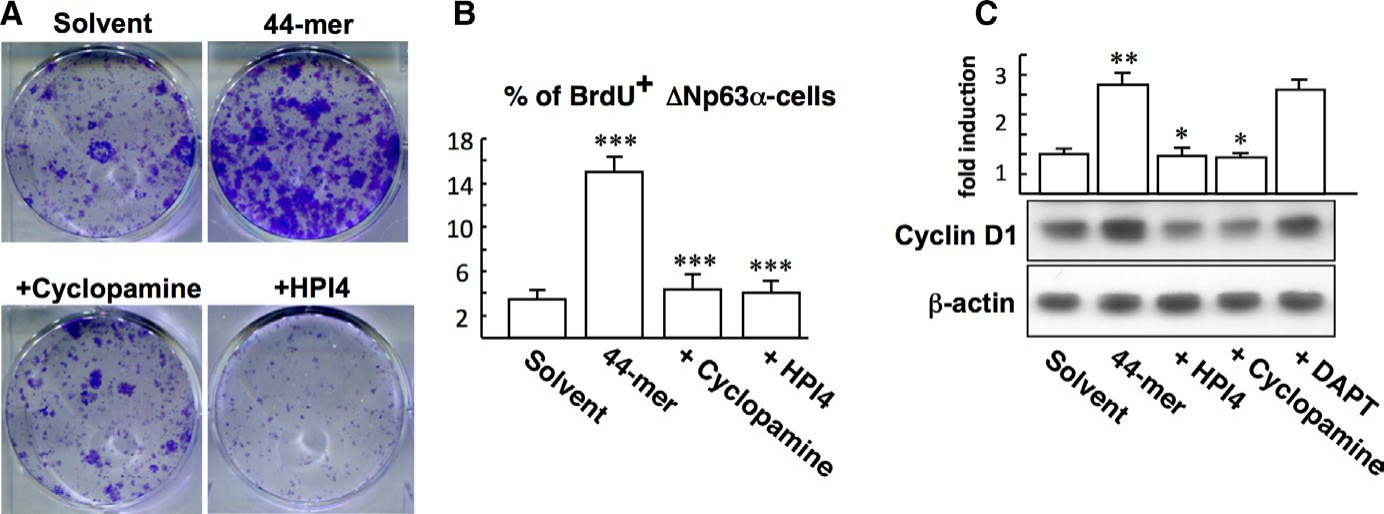
Inhibition of Sonic Hedgehog signalling impedes the proliferation of limbal stem cells (LSC) induced by the 44‐mer. (A) Colony‐forming efficiency assay. LSC were treated with 10 µmol/L 44‐mer in the presence or absence of 1 µmol/L HPI4 or cyclopamine. After 10 days, the colonies were stained by 0.5% crystal violet. These results are representative of triplicate experiments. (B) BrdU labelling assay. LSC were treated with 10 µmol/L 44‐mer in the presence or absence of 10 µmol/L HPI4 or cyclopamine for 24 h. Subsequently, the cells were exposed to 10 µmol/L BrdU for a further 2 h. The percentage of BrdU and ∆Np63α‐double positive cells per total ∆Np63α‐positive cells was calculated. ***P < 0.0002 (44‐mer vs solvent‐treated cells), ***P < 0.001 (44‐mer + Cyclopamine or HPI4 vs 44‐mer‐treated cells) (C) Western blot analysis of the expression of cyclin D1 in LSC which were treated as described above. **P < 0.01 vs solvent‐treated cells. *P < 0.05 vs 44‐mer‐treated cells

To study the role of SHh signalling in 44‐mer‐mediated LSC proliferation, CFE assays were performed with the treatment of 44‐mer, in combination with HPI4 or cyclopamine. The result showed that 44‐mer‐promoted clonogenicity was decreased by HPI4 or cyclopamine (Figure 2A). Cyclopamine and HPI4 also reduced the number of BrdU‐positive LSCs induced by 44‐mer (4.3 ± 1.4% and 4.1 ± 1.1% vs 15.0 ± 1.3%; Figure 2B). In addition, exposure of LSCs to the 44‐mer for 24 hours resulted in a 2.7‐fold increase of the cyclin D1 protein compared to control (*P* = 0.008), and this effect was suppressed by the SHh inhibitors (*P* = 0.001) but not by Notch inhibitor (DAPT) (Figure 2C). These results indicate that 44‐mer‐induced LSC expansion requires activation of SHh signalling.

**Figure 3:**
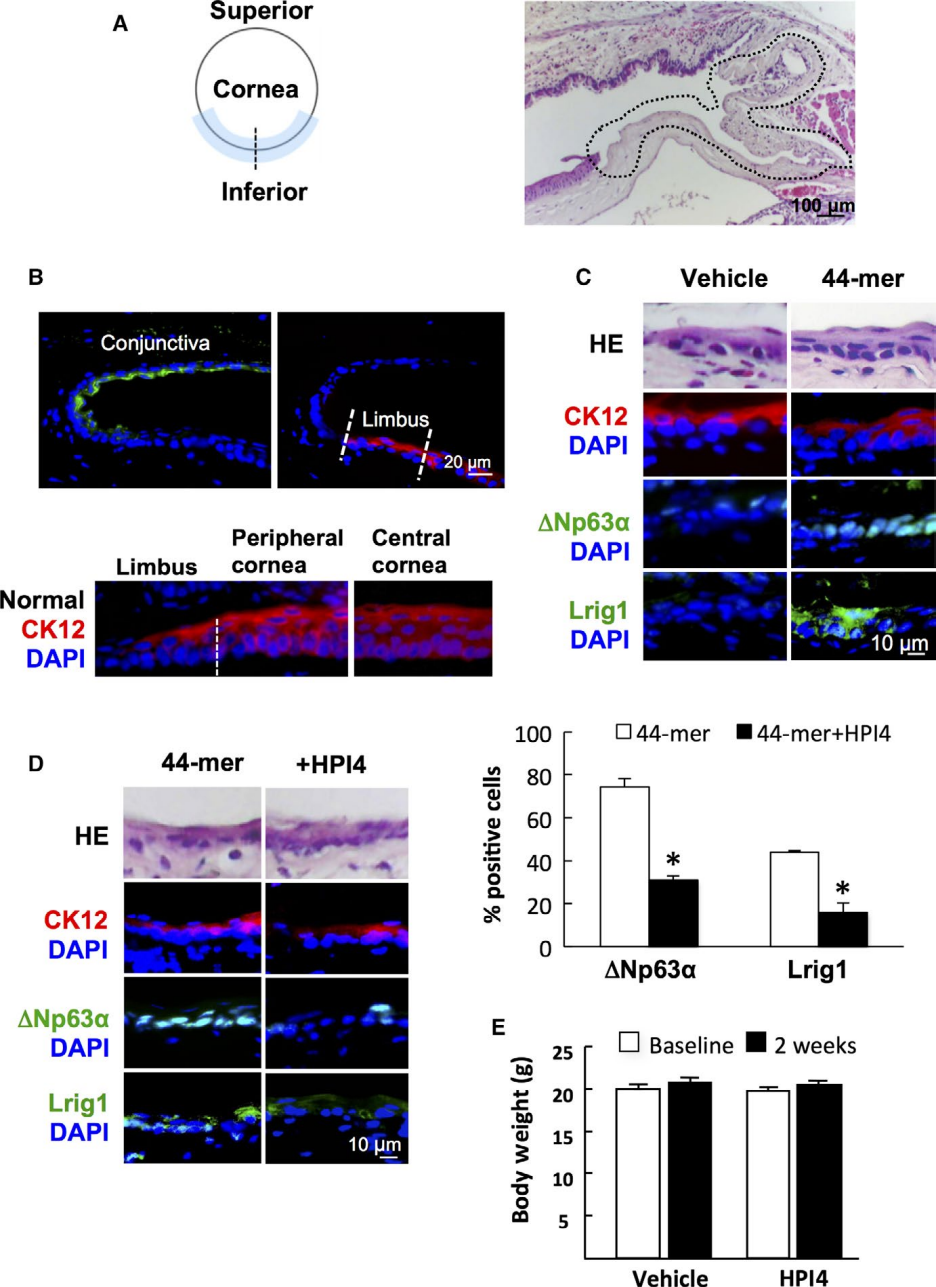
In vivo inhibition of Sonic Hedgehog (SHh) signalling suppressed the 44‐mer effect on limbal epithelial stem cells. (A) Partial limbal surgery. Left panel indicated schematic drawing of the destroyed limbus. Right panel indicated the representative Haematoxylin and eosin section of damaged limbus. The dotted line indicates the destroyed limbal area. (B) Immunostaining of consecutive sections for CK19 and CK12 in mouse normal eyes. (C) Representative micrograph of immunostaining of consecutive sections showed expressions of ∆Np63α and Lrig1 in damaged limbus 2 weeks after treated with the 44‐mer. Representative images are from six eyes of different mice per group. (D) An inhibitor for SHh (HPI4) in addition to 44‐mer was introduced via subconjunctival route. Histogram of immune‐positive cells in limbal area is presented as percentage of the total limbal basal cells. Representative images are from three eyes of different mice per group *P < 0.05 vs 44‐mer‐treated eyes. (E) Mice body weight before and after treatment

To explore the in vivo requirement of SHh signalling for 44‐mer‐induced LSC proliferation, we performed surgical removal of inferior limbal epithelium of mice and treated them with 44‐mer or vehicle (Figure 3A). The presence of LSCs in wounded areas was evaluated by IF. In normal mice, conjunctival epithelium was stained positively with CK19, while corneal and limbal epithelia were stained positively with CK12. Of note, LSCs, which were at basal layer of limbus, were stained negatively with CK12, while whole layer of corneal epithelium was stained positively with CK12 (Figure 3B). The amount of △Np63α‐positive or Lrig1‐positive basal cells was calculated as percentage of the total limbal basal cells. The mice with inferior limbal epithelium removed were evaluated at 2 weeks after injury. The limbal epithelial basal cells in the 44‐mer groups were stained positively with the LSC markers △Np63α (74.26 ± 3.94%) and Lrig1 (43.9 ± 0.87%), while none can be stained in control group (Figure 3C). Furthermore, subconjunctival injection of HPI4 along with 44‐mer suppressed the 44‐mer‐induced ∆Np63α‐positive LSCs by 2.4‐fold decrease and Lrig1‐positive LSCs by 2.8‐fold decrease (Figure 3D). HPI4 injection did not show any severe adverse effect, such as weight loss (Figure 3E). These findings suggest that the 44‐mer enhances restoration of LSCs in the damaged limbus through SHh signalling pathway.

### 3.3 44‐mer induces Gli transcription factors

**Figure 4:**
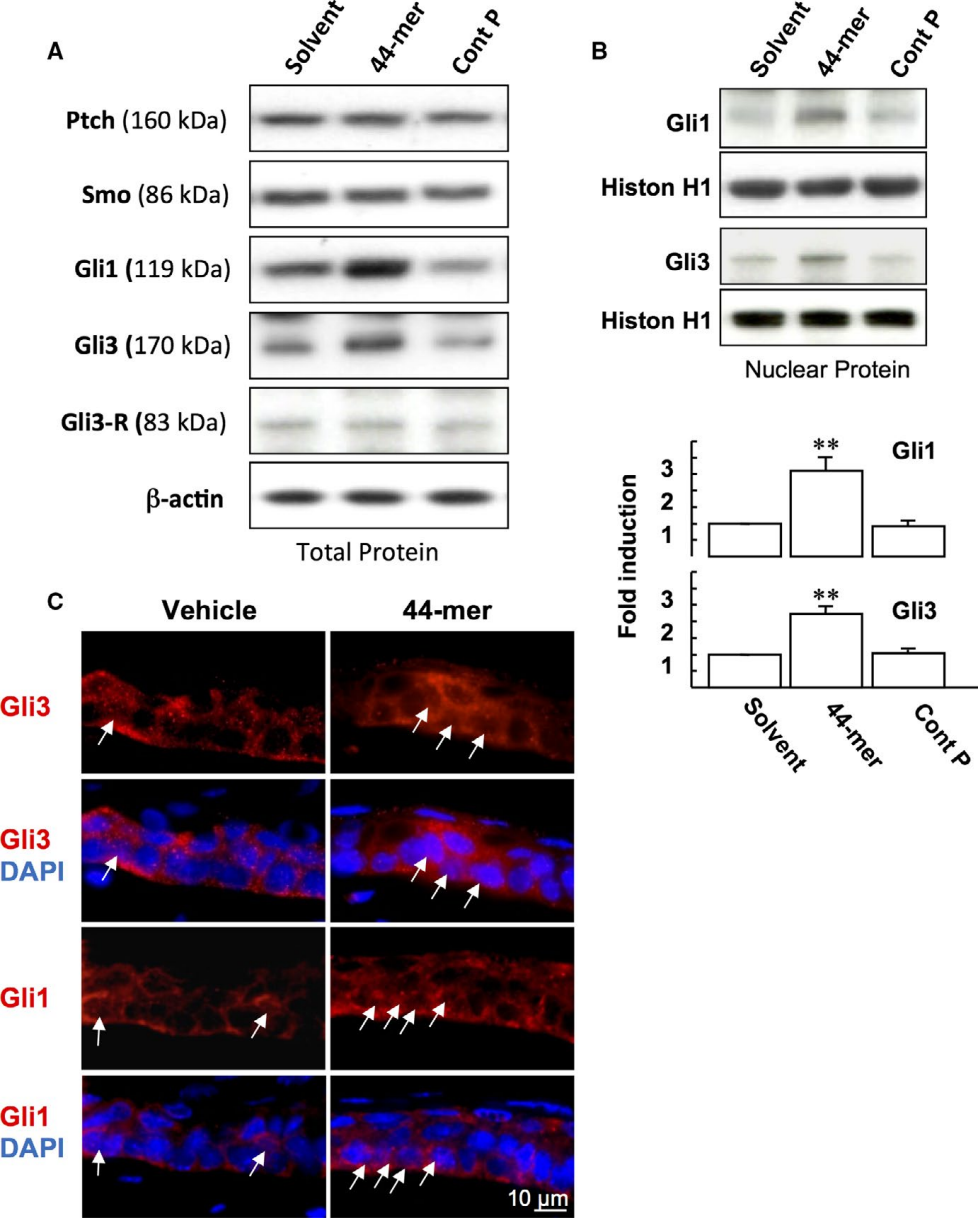
44‐mer induces nuclear level of Gli proteins in limbal epithelial stem cells (LSCs). (A) LSCs were treated with 10 μmol/L 44‐mer or control peptide for 6 h, and expressions of Ptch, SMO, Gli1, Gli3 and Gli‐3R were determined by Western blot analysis. (B) The nuclear levels of Gli1 and Gli3 after the 44‐mer treatment for 6 h were shown. The nuclear fraction was isolated and then subjected to Western blot analysis. The intensities of nuclear Gli1 and Gli3 in the immunoblots were determined by densitometry and normalized to histone H1. **P < 0.01 vs solvent‐treated cells. (C) Representative micrograph of immunostaining of mice eyeball cocultured with 44‐mer for 2 h. Arrows indicated nuclear translocation of Gli1 and Gli3 of LSC at superior limbus

Next, we examined the effect of 44‐mer on the protein expressions of SHh receptors (Ptch and SMO), and Gli transcription factors. We found that after LSCs were stimulated by the 44‐mer for 6 hours, the expressions of Gli1 and full‐length activator of Gli3 (170 kDa) were increased by 3‐fold and 2.7‐fold, respectively, compared to vehicle or control peptide, while the expression levels of Ptch, SMO and Gli3 transcriptional repressor isoform (Gli3‐R) remained unchanged (Figure 4A). The 44‐mer‐induced Gli1 and Gli3 expressions declined to basal levels at 24 hours (N.W.F., T.C.H., Y.P.T., unpublished data). Nuclear translocation of Gli proteins is an indicator of SHh signalling activation. After LSCs were treated with 44‐mer for 6 hours, cell fractionation assays showed an increase of nuclear Gli1 and Gli3 levels compared to vehicle or control peptide‐treated cells (Figure 4B). The mitogenic effect of recombinant SHh on promoting corneal epithelial healing has been reported using organ cultures.^22^ To investigate the activation of SHh signalling, organ cultures of eyeballs with removed inferior limbal epithelium were treated with the 44‐mer. Consistently, nuclear Gli1 and Gli3 levels were increased in the LSCs of superior limbus with 44‐mer treatment (Figure 4C). Taken together, the increase of nuclear Gli1 and Gli3 in LSC following stimulation by 44‐mer suggests that 44‐mer mediates its effect on LSC through activating SHh signalling pathway.

### 3.4 Knockdown of Gli3 impairs the mitogenic activity of 44‐mer on LSCs

**Figure 5:**
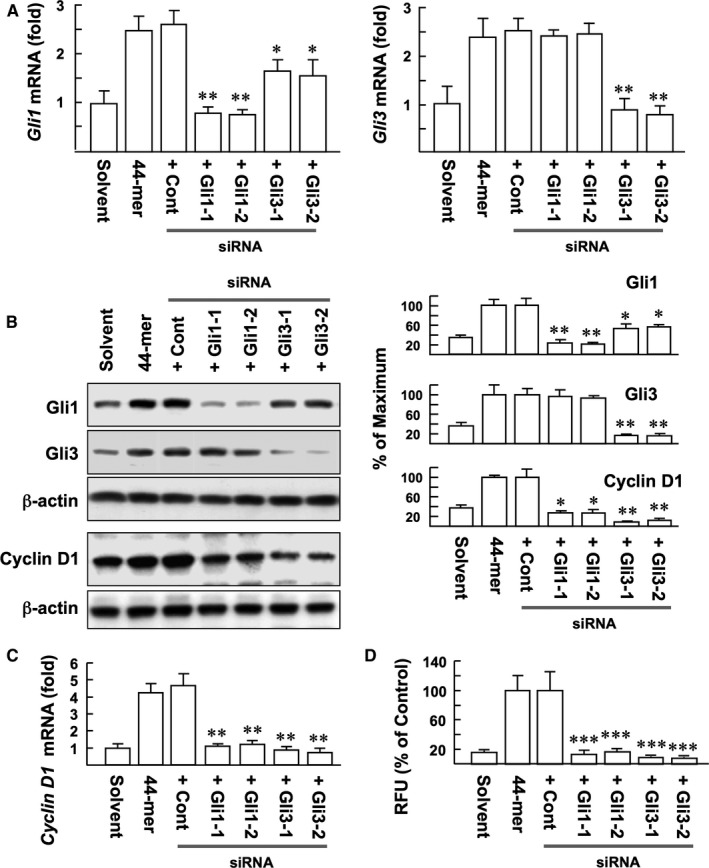
The influence of Gli1 and Gli3 depletion on the mitogenic activity of 44‐mer in LSC. (A) Real‐time PCR analysis of silencing efficacies. The results were normalized to LSC transfected with control siRNA. Average levels of *Gli* mRNA from three repeats of experiments are shown. **P < 0.005 vs control. *P < 0.05 vs control. (B) Western blot analysis. The transfection of LSCs was as described. The expressions of Gli1/Gli3 and cyclin D1 proteins were examined after siRNA transfected LSCs were stimulated by the 44‐mer for 6 and 24 h, respectively. Representative blots and densitometric analysis with the SD from three independent experiments are shown. **P < 0.01 vs control. *P < 0.05 vs control. (C) Relative expression of cyclin D1 determined by real‐time PCR. The values represent the mean ± SD of triplicate qPCRs from three different experiments. **P < 0.006 vs control. (D) Proliferation rates of siRNA transfected LSCs exposed to 44‐mer for 24 h. Results from three repeats of experiments are shown. RFU, relative fluorescence unit; ***P < 0.0001 vs control

To examine whether Gli1 and Gli3 are required for the 44‐mer‐mediated LSC proliferation, we used siRNA to silence the two genes. Real‐time PCR analysis confirmed that Gli‐1 and ‐3 mRNA expressions were significantly inhibited by respective siRNAs. However, knockdown of Gli3 also inhibited 44‐mer‐induced Gli1 expression (Figure 5A, *P* < 0.05 vs control). Western blotting showed that si‐Gli1 led to almost 80% Gli1 protein suppression, and si‐Gli3 significantly suppressed both Gli1 and Gli3 protein levels, by 47% and 83%, respectively (Figure 5B), suggesting that Gli1 expression is partially mediated by Gli3.

It has been demonstrated that cyclin D1 is a target gene of the Gli transcription factors.^18^ Real‐time PCR and Western blotting showed that si‐Gli1 or si‐Gli3 significantly suppressed the ability of 44‐mer to induce cyclin D1 expression, compared to control (Figure 5B,C). Most importantly, si‐Gli1 or si‐Gli3 blocked the 44‐mer‐induced LSC proliferation by 83%‐92%, respectively, compared to control (Figure 5D). Our results indicate that 44‐mer promotes LSC proliferation through Gli1‐ and 3‐mediated cyclin D1 expression.

### 3.5 ATGL activates STAT3 signalling to promote Gli expression

**Figure 6:**
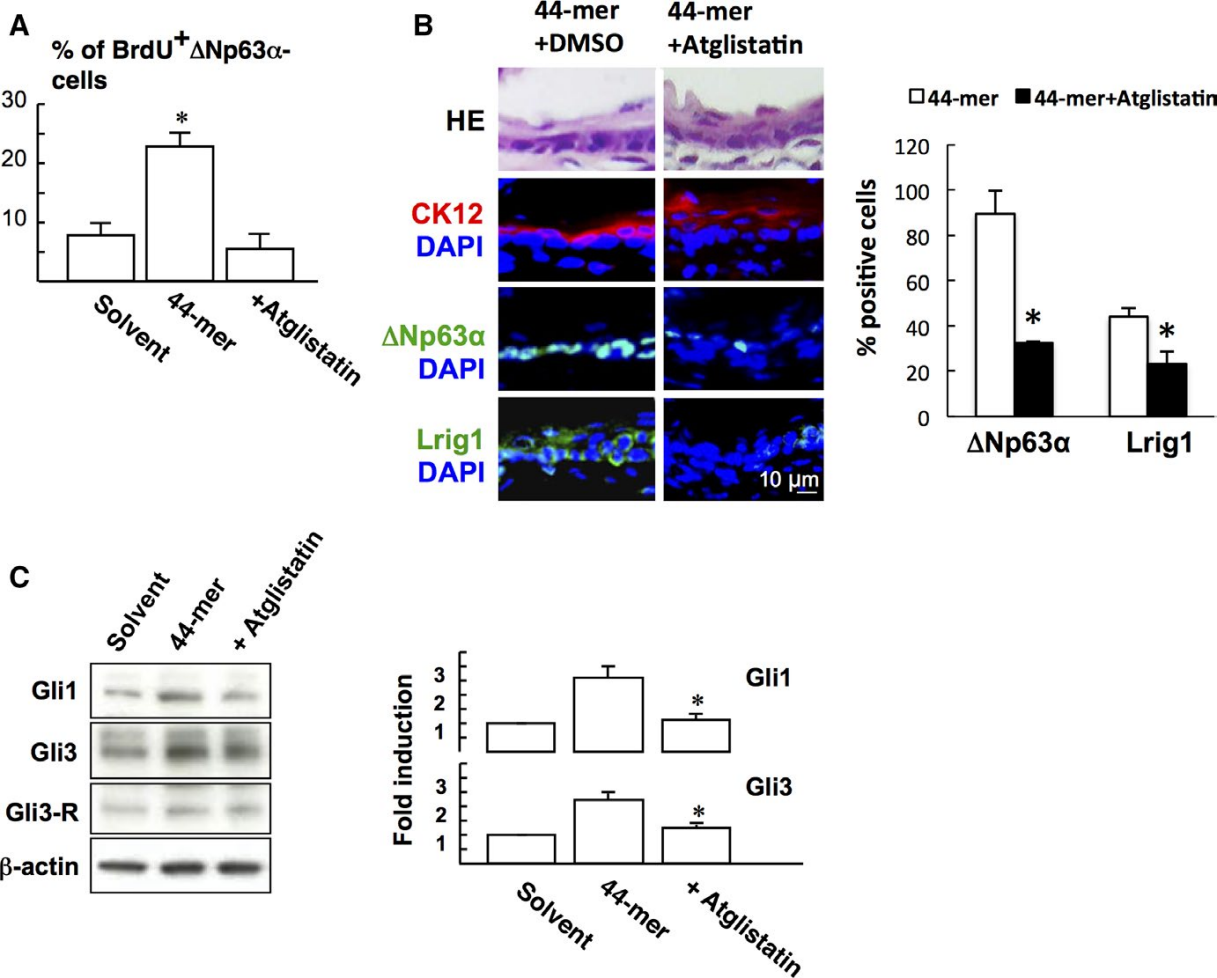
The 44‐mer induces Gli1 and Gli3 expressions via ATGL signalling (A) BrdU‐positive cells were detected by immunofluorescence microscopy as described in Figure 2B. *P < 0.05 vs solvent or adding an inhibitor for ATGL (Atglistatin). (B) Atglistatin in addition to 44‐mer was introduced via subconjunctival route. At 2 weeks, eyeballs were harvested for immunofluorescence staining of consecutive sections for anti‐CK12, anti‐∆Np63α and anti‐Lrig1. Histogram of immune‐positive cells in limbal area is presented as percentage of the total limbal basal cells. *P < 0.05 vs 44‐mer‐treated eyes. Representative images are from three eyes of three mice per group. (C) LSC were treated with the 10 µmol/L 44‐mer for 6 h or pretreated with 10 µmol/L Atglistatin for 1 h and then incubated with the 44‐mer for an additional 6 h. Proteins were detected by Western blot analysis. *P < 0.05 vs 44‐mer‐treated cells

Given that 44‐mer bound to its receptor ATGL to initiates signal transduction,^23^ the role of ATGL in LSC proliferation was investigated. BrdU labelling assay showed that Atglistatin blocked 44‐mer‐induced LSC proliferation in vitro (Figure 6A). In vivo experiment also showed that subconjunctival injection of Atglistatin suppressed the 44‐mer‐induced ∆Np63α‐ (*P* = 0.046) and Lrig1‐positive (*P* = 0.046, Figure 6B) LSC expressions. We further examined the effect of pharmacological inhibition of ATGL signalling on 44‐mer‐induced Gli1 and Gli3 expressions. When LSCs were pretreated with Atglistatin, Gli1 and Gli3 proteins were suppressed to near basal levels (Figure 6C).

**Figure 7:**
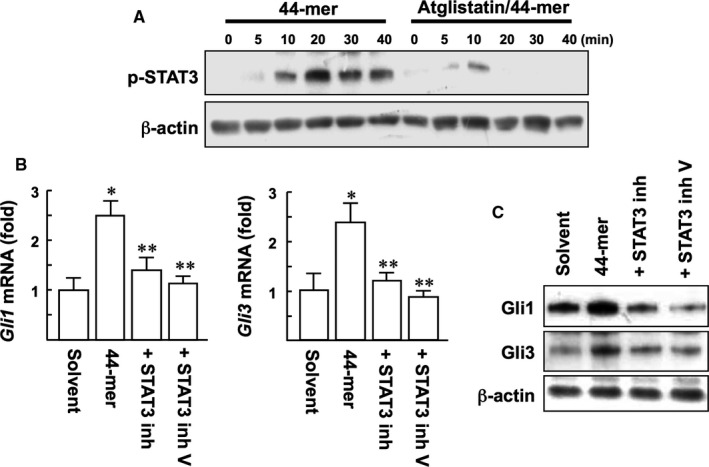
The 44‐mer induces Gli1 and Gli3 expressions via ATGL/STAT3 signalling (A) Limbal stem cells (LSCs) were pretreated with Atglistatin for 1 h prior to stimulation with the 44‐mer. Cells were harvested for Western blot analysis using phospho‐specific antibody to STAT3. (B) Cells were treated with 44‐mer for 3 h or pretreated with 10 µmol/L peptide‐formed STAT3 inhibitor or 1 µmol/L STAT3 inhibitor V for 1 h and then incubated with the 44‐mer for an additional 3 h. The levels of *Gli* mRNA were analysed by PCR and results from three repeats of experiments are shown. *P < 0.05 vs solvent‐treated cells. **P < 0.005 vs 44‐mer‐treated cells. (C) LSCs were exposed to the 44‐mer for 6 h after pretreated with STAT3 inhibitor. Gli proteins were detected by Western blot analysis. Blotting of β‐actin serves as a loading control

PEDF has been shown to induce phosphorylation of STAT3 in LSCs, and PEDF‐ATGL signalling is relied on STAT3 signalling.^14^, ^23^ To determine whether the effect of 44‐mer on SHh activation is dependent on ATGL/STAT3 signalling, pharmacological inhibitors for ATGL and STAT3 were evaluated. Our results showed that Atglistatin suppressed the phosphorylation of STAT3 at Tyr705 in response to 44‐mer (Figure 7A). With the 44‐mer treatment for 3 hours, the mRNA levels of *Gli1* and *Gli3* were significantly up‐regulated by 2.5‐ and 2.4‐fold, respectively, compared to control (Figure 7B). STAT3 inhibitors suppressed the 44‐mer‐induced *Gli1* and *Gli3* mRNA and protein expressions to near basal levels (Figure 7B,C). Collectively, pharmacological inhibition of ATGL/STAT3 signalling attenuated the ability of the 44‐mer to up‐regulate Gli1 and Gli3 expressions.

## 4 DISCUSSION

Damage to the human corneal limbus may lead to permanent dysfunction of the stem cells. Currently, there is no available therapeutics to slow down the progress of LSCD. We have previously demonstrated that LSCs can be expanded by 44‐mer both in vitro and in vivo.^14^, ^16^, ^17^ In this present study, we have further shown that ATGL‐STAT3‐SHh signalling pathway is essentially involved in 44‐mer‐mediated LSC expansion.

The canonical SHh pathway is a stemness pathway which is largely inactive in adult tissue, except for tissue repair and maintenance.^25^ The SHh signal transduction involves binding of processed SHh proteins to their inhibitory receptor Ptch, which leads to activation of the pathway by phosphorylation of SMO.^18^ Subsequently, Gli transcription factors are activated and translocated into the nucleus and modulate the expression of downstream target genes, including cyclin D1, Myc, Bcl2, Bmi1 and Snail, which further regulate cell behaviour.^18^, ^26^ The role of SHh in the LSC population has remained unknown until our current study showing that intrinsic SHh is crucial for LSC self‐renewal. In addition, 44‐mer behaves as an activation signal to interact with SHh/Gli signalling, resulting in modulation of Gli protein expression to promote LSC proliferation. Our study proved that 44‐mer up‐regulates Gli1 and Gli3 in LSCs. We also found that knockdown of *Gli3* reduced Gli1 mRNA and protein levels in 44‐mer‐treated LSCs, suggesting that *Gli1* gene is probably a direct transcriptional target of *Gli3*.^27^ Other signalling molecules, which were reported to be positive regulators of Gli function, include EGF, PDGF, FGF and IGF.^18^, ^21^, ^28^ Although EGF, and FGF promote LSC proliferation,^29^ the interaction between EGF, FGF and SHh‐Gli in LSCs is unknown.

STAT3 is a cytoplasmic protein with Src Homology‐2 domains that act as signal messengers and transcription factors, participating in cellular responses to cytokines and growth factors. PEDF was found to cause a striking activation of STAT3 in myoblasts, hepatocytes and LSCs; pharmacological inhibition of STAT3 blocks PEDF function in these cells.^14^, ^23^, ^30^ Accordingly, in the present study, we found that SHh signalling activated by the 44‐mer was regulated by STAT3 activity as well. These results suggest that cell signalling in response to 44‐mer appears to rely essentially on STAT3 activity. Other well‐studied signalling systems dependent on STAT3 include G‐CSF receptor signalling, HGF and IL‐10.^31^ Apart from promoting SHh signalling activation, the mechanism by which 44‐mer increases LSC population may also involve the direct binding of STAT3 to the △Np63α promoter.^32^ Crosstalk between SHh and STAT3 has been studied in gastric metaplasia, lung adenocarcinoma and skin tumours. All these studies suggest that the interaction between STAT3 and SHh was indirectly mediated through up‐regulation of third‐party factors, such as IL‐6, IL1β, TNFα, IL‐11 and TIF1.^26^, ^33^, ^34^ The precise pathway by which the STAT3 and SHh pathway interacts in LSCs requires further studies.

Various membrane receptors for PEDF have been reported in different cells.^35^, ^36^, ^37^ It was found that ATGL is a putative receptor for PEDF in rabbit corneal epithelial cells.^38^ In our study, either direct inhibition of SMO by cyclopamine,^26^ or inhibition of downstream of SMO by HPI4,^39^ abolished the 44‐mer‐induced LSC expansion. A recent report indicated that SHh activates phospholipase A2 to release arachnoid acids, which promotes SMO ciliary accumulation and signalling.^40^ Because PEDF can regulate triglyceride metabolism in hepatocytes through ATGL,^41^ one may speculate that PEDF augments the function of SMO to enhance the expression of downstream Gli proteins.

The restoration of LSC population in the limbal wound promoted by the 44‐mer is probably because of the migration of LSCs, either from lateral cell displacement or the central zone.^16^ The target genes of the SHh pathway responsible for epithelial‐mesenchymal transition^26^ may contribute to LSC migration. On the other hand, a scenario of one‐for‐one replacement from neighbouring LSCs, stimulated by death of LSCs,^42^ is another possible mechanism in which symmetrical division could be promoted by the 44‐mer. Our study progresses the LSC biology field by providing clues on the molecular programs that orchestrate LSC activity. Exogenous cues, such as PEDF, which modify the signals that determine stem‐cell fate decisions, have the translational potential for the treatment of LSCD.

## CONFLICT OF INTEREST

Patent: Use of PEDF‐derived polypeptides for promoting stem cells proliferation and wound healing (T.‐C. Ho, Y.‐P. Tsao). Nai‐Wen Fan and Cheng‐Wen Wu: none.

## AUTHOR'S CONTRIBUTION

Nai‐Wen Fan and Tsung‐Chuan Ho performed the research and analysed the data. Nai‐Wen Fan, Tsung‐Chuan Ho, Cheng‐Wen Wu and Yeou‐Ping Tsao designed the research. Yeou‐Ping Tsao contributed essential reagents or tools. Nai‐Wen Fan and Tsung‐Chuan Ho wrote the paper. Cheng‐Wen Wu and Yeou‐Ping Tsao revised the paper.

## DATA AVAILABILITY STATEMENT

The datasets used and/or analysed during the current study are available from the corresponding author on reasonable request.
